# A pharmacological approach in newly established retinal vein occlusion model

**DOI:** 10.1038/srep43509

**Published:** 2017-03-02

**Authors:** Shinichiro Fuma, Anri Nishinaka, Yuki Inoue, Kazuhiro Tsuruma, Masamitsu Shimazawa, Mineo Kondo, Hideaki Hara

**Affiliations:** 1 Molecular Pharmacology, Department of Biofunctional Evaluation, Gifu Pharmaceutical University, Gifu, Japan; 2 Department of Ophthalmology, Mie University Graduate School of Medicine, Tsu, Japan

## Abstract

The mechanism underlying the effects of anti-vascular endothelial growth factor (VEGF) antibody in retinal vein occlusion (RVO) treatment is poorly understood, partly due to the lack of RVO animal models that mimic clinical pathology. The aims of this study were to establish a suitable RVO model, clarify the pathogenic mechanisms, and evaluate the effects of anti-VEGF antibody in the model. Mouse retinal veins were occluded by laser photocoagulation after rose bengal injection. Reduction of the b/a wave amplitude ratio, retinal nonperfusion, cystoid edema, and hard exudates were observed after occlusion, and expression of RVO-related genes was altered. Administration of anti-VEGF antibody immediately, or 7 days, after occlusion resulted in reduction and increase of the nonperfused area, respectively. We conclude that the present model will be useful for clarification of the pathogenic mechanisms, and that the timing of anti-VEGF antibody administration is important for the successful amelioration of retinal nonperfusion.

Retinal vein occlusion (RVO) is the second most common retinal vascular disorder in developed countries after diabetic retinopathy and causes haemorrhage and edema, leading to painless loss of vision^1^. RVO can be classified as one of two types: branch retinal vein occlusion (BRVO), where any branch retinal vein is occluded, and central retinal vein occlusion (CRVO), where the main vein in the retina is blocked. BRVO is about five times more common than CRVO and usually occurs at sites where arteries intersect with veins^2^. Although the number of patients with RVO is large (approximately 16 million people), available treatments for the macular edema associated with RVO are suboptimal^3^. Indeed, ophthalmologists have been using anti-vascular endothelial growth factor (VEGF) antibodies, such as bevacizumab or ranibizumab, as off-label treatments for patients with RVO. Recently, the Food and Drug Administration approved ranibizumab for treatment of RVO based on randomized clinical trial evidence. However, treatment with anti-VEGF antibody has some limitations, due to the lack of understanding of its mechanism of action. For example, some patients with RVO do not respond to treatment with bevacizumab or ranibizumab, while others experience disease recurrence. In addition, the cost of this type of treatment is extremely high^4^,^5^. Furthermore, the effects of anti-VEGF antibodies on retinal nonperfusion are poorly understood. Conflicting reports indicate that administration of anti-VEGF antibody can both reduce^6^,^7^ and enlarge^8^,^9^ the size of the retinal nonperfusion area, which has led to confusion among ophthalmologists. Therefore, strategies to elucidate the mechanism of action of anti-VEGF antibodies or explore new therapies exploiting targets other than the VEGF pathway are imperative. However, novel medicines for patients with RVO are lacking, and the mechanism of action of anti-VEGF therapy is not well understood in RVO. One reason for these limitations is the lack of an effective RVO animal model exhibiting characteristics similar to clinical manifestations such as cystoid edema and retinal nonperfusion. Cystoid edema is a type of edema frequently observed in the inner nuclear layer (INL), due to destruction of the outer blood-retinal barrier and vascular leakage in patients with diabetic retinopathy, intraocular inflammation, and RVO^10^,^11^. Retinal nonperfusion, which can be classified into two types, ischemic and non-ischemic, according to the size of the nonperfused retina, is also frequently observed in patients with RVO^12^.

Many studies using RVO animal models (monkey, rat, rabbit, and mouse) have been reported^13^,^14^; however, these previously reported animal models have important limitations. First, they do not exhibit cystoid edema, which is one of the most important symptoms of RVO, since it is strongly associated with visual impairment. Patients who have severe or chronic cystoid edema (>8 months) have permanent diminution of vision, secondary to disruption of intraretinal connections^15^, and some exhibit severe cystoid edema accompanied by neovascular glaucoma^16^,^17^,^18^. Hence, it is important that an experimental model of RVO exhibits cystoid edema. The second limitation of previous RVO models is that they undergo spontaneous recanalization, with some reports of recanalization within only 3 days after occlusion, resulting in the improvement of retinal function without treatment19,20. Therefore, it is difficult exactly to evaluate some compounds using previous models which can be administrated only within 3 days because there are some cases to administrate candidate compounds during more than 3 days. The third limitation is laser-induced inflammation. It is possible to induce retinal edema (swelling) via laser-induced inflammation. Established RVO models show destruction of the outer retinal layer and collateral damage affecting the photoreceptors in laser irradiated-areas. In summary, previous RVO experimental models have a number of limitations, resulting in a lack of success in elucidation of the pathogenic mechanisms of RVO and discovering novel treatment strategies.

The aims of this study were to establish an RVO mouse model mimicking its clinical symptoms, to clarify the pathogenic mechanisms underlying RVO, and to evaluate the effects of anti-VEGF antibody on edema and retinal nonperfusion.

## Results

### RVO Model

RVO models were developed by occlusion of three retinal veins per eye in BALB/c, C57BL/6J and ddY mice, by performing laser photocoagulation according to an established method with some modifications^13^,^21^ (see methods section for details).

### Cystoid edema and haemorrhage were observed in the experimental RVO model

**Figure 1:**
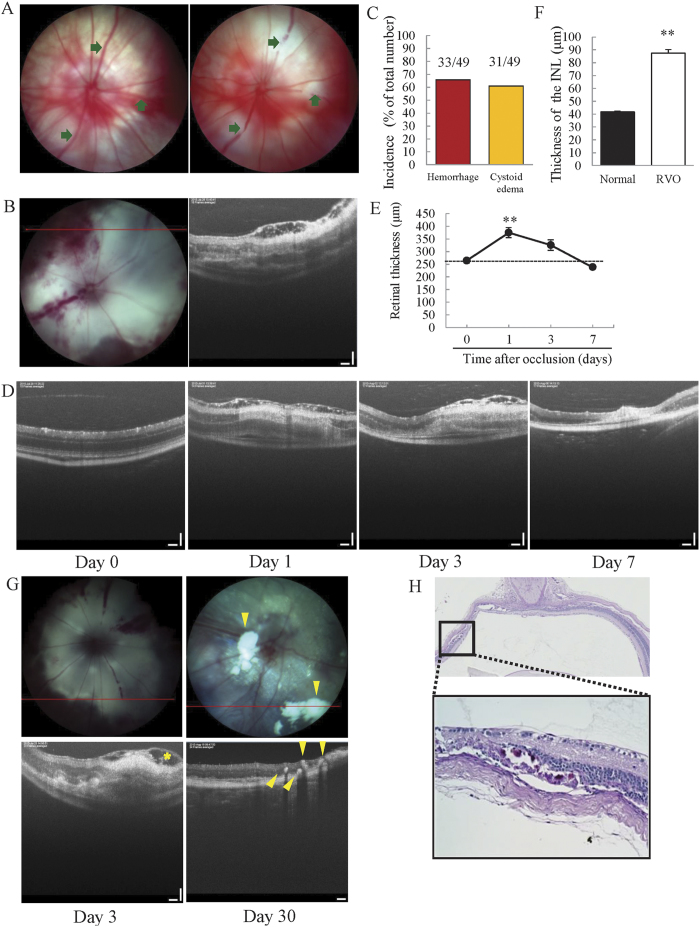
Both retinal haemorrhage and cystoid edema were observed in the experimental mouse model of RVO. (**A**) Images of a non-laser irradiated retina (left) and a retina after laser irradiation (right). The arrows indicate occluded sites. (**B**) Fundus photography image (left) and OCT image (right), taken 3 days after photocoagulation. Haemorrhage and cystoid edema were observed (n = 49). (**C**) The incidences of haemorrhage and cystoid edema were 65.8% and 60.9%, respectively. (**D**) OCT images taken 0, 1, 3, and 7 days after photocoagulation. Cystoid edema was observed on days 1 and 3. (**E**) The plot below illustrates quantitative retinal thickness data. Retinal thickness was significantly increased on day 1 compared to day 0 and gradually recovered over the course of the experiment. Data are expressed as means ± S.E.M (n = 4–7). ^##^*P* < 0.01 vs. day 0 (Dunnett’s test). (**F**) Quantitative analysis of INL thickness 1 day after occlusion. INL thickness was significantly increased 1 day after occlusion. Data are expressed as means ± S.E.M (n = 4–7). ^##^*P* < 0.01 vs. day 0 (Student’s *t-*test). (**G**) RVO mice have white flecks (fundus photography) and hyper-reflective dots (OCT) in the INL and OPL. Hyper-reflective dots were present in the same areas where severe edema developed. Arrow head indicates hard exudates. (**H**) PAS-positive deposits were located in the INL and OPL. Scale bar represents 50 μm.

Temporal alterations in the retinal vasculature and retinal layer were examined by retinal imaging microscopy and optical coherence tomography (OCT) imaging. Occlusion-induced ischemia caused the formation of cystoid edema and haemorrhage in ddY mice. Cystoid edema was not observed in BALB/c and C57BL/6J mouse strains, although the technique used for occlusion of retinal veins was the same as that being used for ddY mice (Supplemental Fig. 1); therefore, all remaining experiments used only ddY mice. The incidence of haemorrhage and cystoid edema was 65.8% and 60.9%, respectively in ddY mice (Fig. 1B,C). Swelling edema was observed 1 day after occlusion, and cystoid edema was present in the retinal nerve fibre layer (RNFL) (Fig. 1D). The swelling and cystoid edema were observed until day 3. We quantified the thickness of retinas to investigate time-dependent changes in retinal thickness. Retinal thickness increased dramatically 1 day after occlusion, and then gradually recovered over time. On day 7, the thickness of retinas had returned to that observed on day 0 (Fig. 1E). White flecks and hyper-reflective dots (hard exudates) were observed in the majority of occluded eyes (90%) and these were primarily located in the outer plexiform layer (OPL) 30 days after occlusion, in the same areas where edema was observed on day 3 (Fig. 1G). Moreover, periodic acid-Schiff (PAS)-positive deposits were identified in the inner nuclear layer (INL) and OPL (Fig. 1H).

### Cystoid and swelling edema were presence in the INL and OPL, but not the ONL

**Figure 2:**
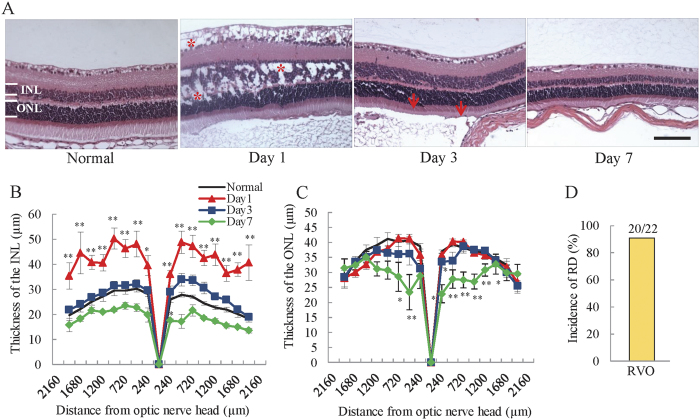
Edema was located mainly in the inner layer of the retina. (**A**) Representative images of H&E-stained retinas. Asterisk (_*_) indicates cystoid edema. Scale bar = 50 μm. Cystoid edema was observed 1 day after photocoagulation in the INL and ONL. Plots below illustrate quantitative INL (**B**) and ONL (**C**) thickness data. The thickness of the INL was dramatically increased 1 day after occlusion, gradually recovering in a time-dependent manner. ONL thickness was significantly decreased on day 7, and there was no change on days 1 and 3. The data are expressed as means ± S.E.M. (n = 3–7). ^##^*P* < 0.01, ^#^*P* < 0.05 vs. untreated control (Dunnett’s test). INL; inner nuclear layer; ONL; outer nuclear layer. (**D**) The incidence of retinal detachment (arrow) was approximately 90.9% (20/22).

To clarify which retinal layers are thickened by occlusion-induced ischemia, we quantified the thickness of the outer nuclear layer (ONL) and INL within the H&E-stained retina. In the RVO mouse retina, the thickness of the INL was remarkably increased 1 day after occlusion, and improved on day 3 (Fig. 2A,B). Seven days after photocoagulation, the thickness of the INL was decreased compared to untreated mice. In contrast, the ONL remained unchanged in thickness on days 1 and 3, with a reduction in thickness on day 7 (Fig. 2C). Furthermore, cystoid edema was observed in the RNFL, INL, and OPL on day 1 but was not present on days 3 and 7 (asterisks, Fig. 2A). Moreover, retinal detachment was observed in approximately 90% of animals 1 day after occlusion (Fig. 2A arrows, 2D).

### Occlusion caused a deterioration of visual function and a decrease in the b/a wave amplitude ratio

**Figure 3:**
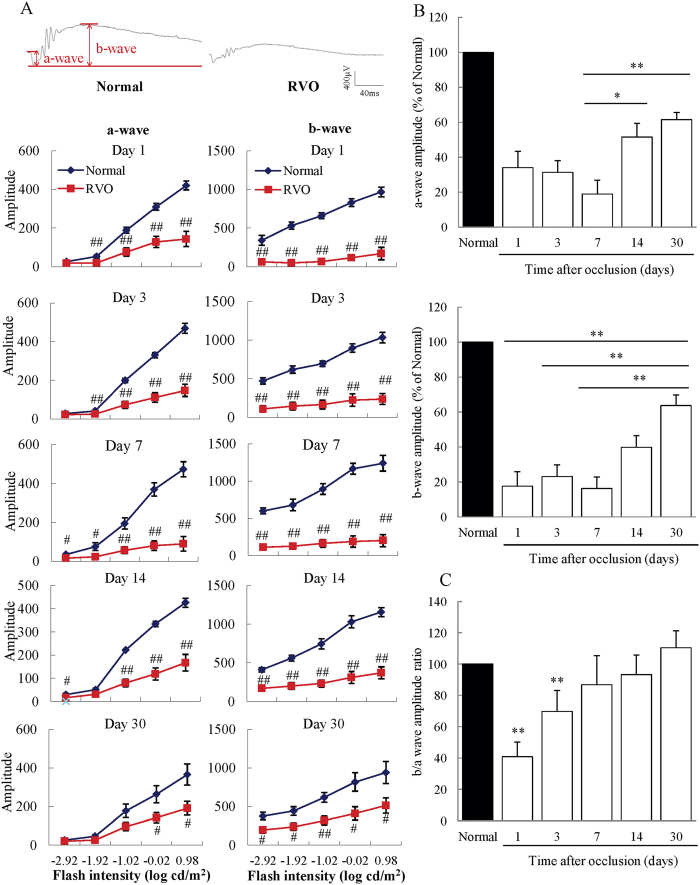
Significantly decreased b/a wave amplitude ratios in the RVO model. (**A**) From days 1 to 30, both the a- and b-waves were dramatically decreased in RVO mice compared to untreated controls. Data are expressed as means ± S.E.M. (n = 3–7). ^##^*P* < 0.01, ^#^*P* < 0.05 vs. untreated controls (Student’s *t*-test). (**B**) The decreased a-wave amplitude recovered significantly on days 14 and 30, compared to those on day 7. Moreover, the reduced b-wave amplitude recovered on day 30 compared to days 1, 3, and 7. Data are expressed as means ± S.E.M. ^**^P < 0.01, ^*^P < 0.05 (one-way ANOVA followed by Bonferroni’s post hoc comparison test). (**C**) The b/a wave amplitude ratio was significantly decreased on days 1 and 3 compared to untreated controls. The decreased b/a wave ratio recovered gradually and returned to a normal level by day 30. Data are expressed as means ± S.E.M. ^**^P < 0.01, ^*^P < 0.05 vs. untreated controls (Student’s *t-*test).

To investigate temporal changes in retinal function after occlusion, we performed electroretinography (ERG) on RVO mice on days 1, 3, 7, 14, and 30. Both a- and b-waves were remarkably reduced compared to untreated mice at each time point (days1 to 30); however, they demonstrated gradual recovery after day 7 (Fig. 3A). In particular, the a-wave amplitude showed statistically significant increases on days 14 and 30 compared to day 7, and the b-wave amplitude was significantly elevated on day 30 compared to days 1, 3, and 7 (Fig. 3B). To assess the relative function of the inner retina as compared to the outer retina, we calculated the b/a wave amplitude ratio at each time point and found that it was significantly reduced on days 1 and 3 compared to untreated mice (Fig. 3C), with the ratio of RVO mice being only 40.9% that of untreated mice on day 1. The decreased b/a wave amplitude in treated animals gradually returned to the normal range by day 30.

### RVO mice developed retinal nonperfusion, which reduced in extent over time

**Figure 4:**
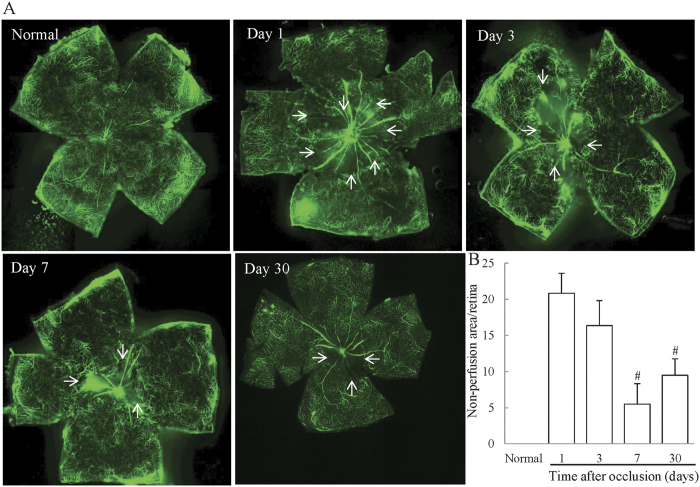
Retinal nonperfusion in the RVO model mouse. (**A**) Representative images of flat-mounted retinas in untreated and RVO mice on days 1, 3, 7, and 30. Arrows = boundary of non-perfused area (**B**) Evaluation of the area of retinal nonperfusion using ImageJ software, demonstrating the development of retinal nonperfusion on days 1, 3, 7, and 30 after occlusion. The nonperfused areas on days 7 and 30 were significantly reduced compared to those on day1. Data are expressed as means ± S.E.M. (n = 4–6). ^*^P < 0.05 vs RVO mice on day1 (one-way ANOVA followed by Bonferroni’s post hoc comparison test).

To confirm whether retinal nonperfusion was induced over time, we prepared flat-mounted retinas of RVO mice, and visualized and quantified the size of nonperfused areas. No retinal nonperfusion was observed in untreated mice, whereas retinal nonperfusion was observed on days 1, 3, 7, and 30 in RVO mouse retinas (Fig. 4A). Quantification of the size of nonperfused areas as a proportion of the whole retina using ImageJ software was performed to clarify time-dependent changes in retinal nonperfusion. On day 1, the nonperfused area accounted for 20.8% of the retina, gradually reducing to 16.3% on day 3. On days 7 and 30, nonperfused areas were 8.9% and 9.4%, respectively, representing significant decreases compared to day 1 (Fig. 4B).

### The decrease of blood flow was maintained for 30 days

**Figure 5:**
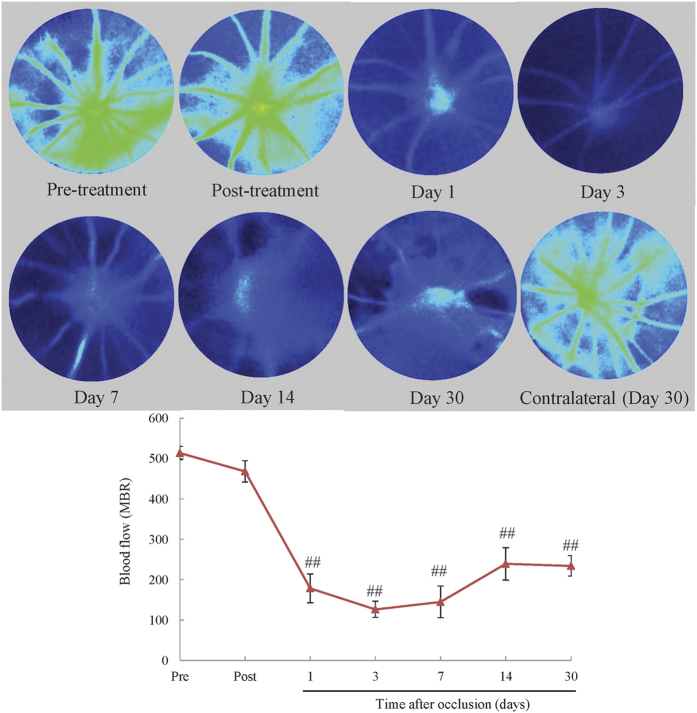
The reduction of blood flow persisted until day 30. Blood flow was measured by LSFG before and immediately after occlusion, and on days 1, 3, 7, 14, and 30 to verify the duration of the reduction of blood flow. Blood flow was significantly reduced compared to the pre-treatment group 1, 3, 7, 14, and 30 days after occlusion. The data are expressed as means ± S.E.M. (n = 5–8). ^##^*P* < 0.01 vs pre-treatment group (Dunnett’s test).

We examined the retinal blood flow by Laser Speckle Flowgraphy immediately and 1, 3, 7, 14, and 30 days after occlusion. Blood flow was minor reduction immediately after occlusion, while on days 1, 3, 7, 14, and 30, it was significantly reduced compared to the pre-treatment group, albeit the blood flow of contralateral retina was unchanged 30 days after occlusion (Fig. 5).

### The expression of RVO-related and inflammatory genes was altered in the RVO mouse model

**Figure 6:**
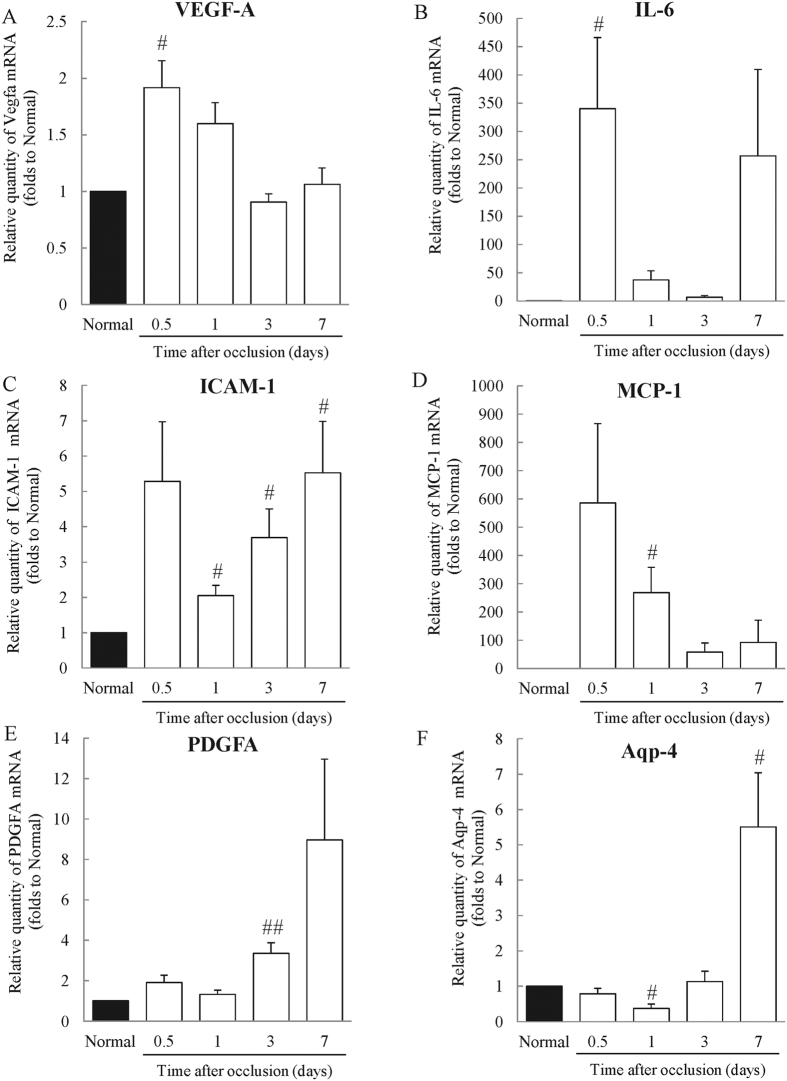
Expression of retinal vein occlusion-related genes in the RVO model. The expression of RVO-related and inflammatory genes were evaluated by real-time PCR analysis 0.5 (12 h), 1, 3, and 7 days after occlusion and in untreated mice. (**A**) The expression level of *Vegfa* mRNA was significantly increased 12 h after occlusion, but unchanged compared to untreated mice after 3 and 7 days. (**B**) The expression level of *Il6* mRNA was remarkably increased 12 h after occlusion. On days 1, 3 and 7, there was no statistically significant difference in expression levels compared to untreated controls. (**C**) The expression level of *Icam1* mRNA was significantly augmented on days 1, 3, and 7. (**D**) The expression level of *Mcp-1* was remarkably increased, to approximately 260 times that of untreated controls. Twelve hours after photocoagulation, the difference was not statistically significant. (**E**) The expression level of *Pdgfa* mRNA was increased to three times or more that of untreated mice. (**F**) The expression level of *Aqp4* was decreased on day 1; however, it was increased to approximately five times that of untreated mice by day 7. Data are expressed as means ± S.E.M. (n = 3–5). ^##^*P* < 0.01, ^#^*P* < 0.05 vs. Normal (Student’s *t*-test).

To confirm whether RVO-related (vascular endothelial growth factor A, *Vegfa*; aquaporin 4, *Aqp4*) and inflammatory (interleukin-6, *Il6*; intercellular adhesion molecule 1, *Icam1*; Monocyte Chemoattractant Protein-1, *Mcp-1*; platelet derived growth factor alpha, *Pdgfa*) mRNAs were expressed in retinas in the experimental model, we performed real-time reverse transcription (RT)-PCR analysis. The expression of *Vegfa* was increased compared to untreated mice 12 h after photocoagulation; however, there was no change in *Vegfa* expression between untreated and RVO mouse retinas on days 3 and 7 (Fig. 6A). For *Il6*, expression levels appeared elevated 12 h and 1, 3, and 7 days after photocoagulation compared to untreated mice (Fig. 6B). The expression of *Icam1* was remarkably increased at 12 h and on days 1, 3, and 7 after photocoagulation (Fig. 6C). *Mcp-1* expression was increased 12 h and 1 day after occlusion compared to untreated mice, although the difference was not statistically significantly at 12 h (Fig. 6D). The expression of *Pdgfa* was also increased 3 days after photocoagulation compared to untreated controls (Fig. 6E). *Aqp4* expression was significantly reduced 1 day after occlusion; however, after 7 days, its expression was increased compared with untreated controls (Fig. 6F).

### Anti-VEGF antibody ameliorated edema in the INL

**Figure 7:**
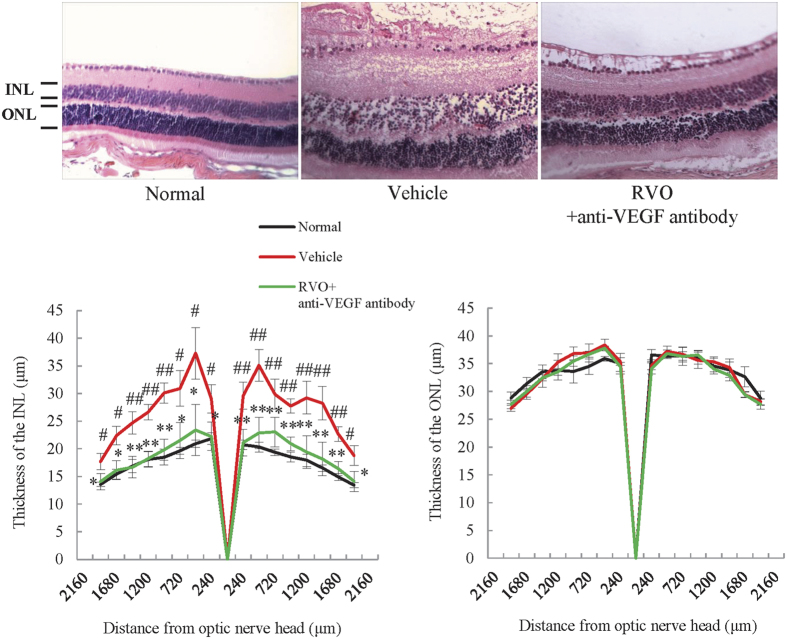
Anti-VEGF antibody ameliorated cystoid and swelling edema in the INL. Representative images of H&E-stained retinas from untreated, RVO, and RVO + anti-VEGF antibody treated groups on day 1. Plots below illustrate quantitative INL and ONL thickness data. The thickness of the INL was dramatically increased 1 day after occlusion and this was prevented by the treatment with anti-VEGF antibody. ONL thickness was not changed by laser irradiation or treatment with anti-VEGF antibody. Data are expressed as means ± S.E.M. (n = 4–5). ^*^P < 0.05 (one-way ANOVA followed by Bonferroni’s post hoc comparison test).

Although anti-VEGF therapy has frequently been used to treat RVO in clinical practice, there are no reports of the use of anti-VEGF antibody to demonstrate the validity of a RVO model. Therefore, we used mouse anti-VEGF antibody to determine whether this type of treatment is able to ameliorate the increased thickness of the INL in the RVO model. As expected, intravitreal administration of anti-VEGF antibody immediately after occlusion reduced the thickness and decreased cystoid edema in the INL of RVO mice. By contrast, anti-VEGF antibody did not affect the thickness of the ONL (Fig. 7).

### Treatment of RVO mice with anti-VEGF antibody in the early phase after occlusion reduced the area of retinal nonperfusion, but led to enlargement when administered at a later time point

**Figure 8:**
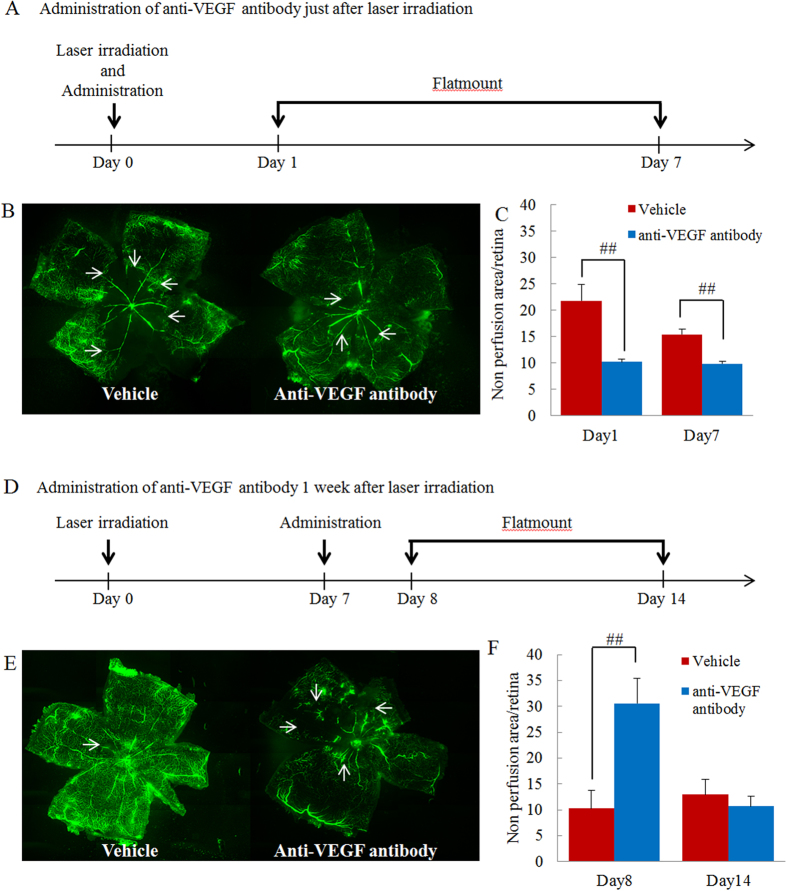
Anti-VEGF antibody prevented progression of retinal nonperfusion when administered in the early phase after occlusion but aggravated nonperfusion when administered in the late phase. (**A**) Protocol of early phase administration. Anti-VEGF antibody was intravitreally administered immediately after occlusion. Sampling was performed 1 and 7 days after administration. (**B**) Representative images of flat-mounted retinas (vehicle and anti-VEGF antibody treated groups). (**C**) Illustration of quantitative retinal nonperfusion area data. Nonperfused areas were reduced 1 and 7 days after occlusion. Data are expressed as means ± S.E.M. (n = 4–8). ^##^*P* < 0.01 (Student’s *t*-test). (**D**) Protocol of late phase administration. Anti-VEGF antibody was administrated intravitreally 7 days after occlusion. Sampling was performed 1 and 7 days after administration. (**E**) Representative images of flat-mounted retinas (vehicle and anti-VEGF antibody treated groups). (**F**) Quantitative illustration of retinal nonperfusion area data. The nonperfusion area was increased by anti-VEGF antibody 1 day after administration but was unchanged 7 days after administration. Data are expressed as means ± S.E.M. (n = 4–5). ^##^*P* < 0.01 (Student’s *t*-test).

There have been opposing reports about the effects of anti-VEGF antibody on the area of retinal nonperfusion in clinical practice. In this study, anti-VEGF antibody was administrated either immediately or 7 days after occlusion, to investigate the effect on the area of retinal nonperfusion. Administration of anti-VEGF antibody immediately after occlusion led to a significant reduction in the nonperfused area 1 and 7 days after administration (Fig. 8A–C). In contrast, administration of anti-VEGF antibody 7 days after occlusion led to an enlargement of the size of the nonperfused area 1 day after administration, with no change apparent after 7 days (Fig. 8D–F).

## Discussion

In this study, we investigated the effects of anti-VEGF antibody on edema and retinal nonperfusion in a newly established RVO animal model with many characteristics comparable to those observed in clinical practice, including cystoid edema, haemorrhage, retinal nonperfusion, and hard exudates. First, we observed the ocular fundus and retinal layer using a Phoenix fundus microscope and OCT to determine whether haemorrhage and cystoid edema occurred in the mouse model. Both cystoid edema (31/49) and haemorrhage (33/49) were observed at 1 and 3 days after occlusion, respectively. Both haemorrhage and cystoid edema are frequently observed in patients with RVO^22^. Intraocular haemorrhage is a common occurrence in certain chronic retinal diseases, including diabetic retinopathy and RVO, and results from an increase in retinal vascular permeability and leukostasis due to breakdown of the blood-retinal barrier^22^,^23^,^24^. Hence, retinal haemorrhage is one of the most important symptoms of RVO. Cystoid edema was also observed 1 day after occlusion in the RVO mouse model by OCT and histological staining. Using OCT, cystoid edema was only observed in the RNFL, whereas H&E staining of the retina revealed cystoid edema in the RNFL, INL, and OPL albeit the thickness of INL was significantly increased. These differences may be a consequence of the limitations of the resolution achieved using OCT imaging. A previous clinical study indicated that cystoid macular edema was present in the ONL, OPL, INL, inner plexiform layer, ganglion cell layer and RNFL of 30.8%, 77.9%, 77.9%, 36.9%, 48.8% and 4.9% of eyes, respectively^25^, suggesting that cystoid edema was mainly present in the OPL and INL. In the present experimental RVO model, cystoid edema was also predominantly observed in the OPL and INL. Cystoid edema was not observed in other mouse strains (BALB/c and C57BL/6 J mice), despite irradiation of retinal veins under the same conditions as those used for ddY mice (Supplemental Fig. 1), consistent with previous reports that vasculature and reactiveness against ischemia differs among mouse strains, and demonstrating that the choice of strain is important^26^,^27^. However, mice do not have macula, the primary site of cystoid edema formation in RVO patients. This is a serious disadvantage of the present model. To establish a model more similar to patients, non-human primate models are strongly recommended. Hence, the characteristics of the present model were not identical to those of patients; nevertheless, our model does demonstrate more similar characteristics than those of previous reported models.

Retinal swelling edema, a type of edema that causes cytotoxicity, was also observed in our model. Previous reports indicate that this swelling is caused by alterations in expression of *Aqp4*^28^,^29^. Aquaporins are transmembrane water channels that conduct water bidirectionally via transcellular osmotically driven fluid movement^30^. In pathological situations, such as ischemia, altered *Aqp4* expression induces the accumulation of intracellular K^+^ ions, resulting in a change in the osmotic gradient between the extracellular space and Müller cells and consequent swelling edema^30^,^31^. In this experimental RVO model, both the formation of swelling edema and decreased *Aqp4* expression were observed, indicating that the retinal swelling is likely to be caused by downregulation of *Aqp4*.

To evaluate the effect of retinal venous occlusion on retinal function, we performed ERG analysis 1, 3, 7, 14 and 30 days after photocoagulation. The retinal origins of the a- and b- waves are not precisely determined, but they are believed to originate mainly from photoreceptors and bipolar cells, respectively. The amplitudes of both a- and b- waves were significantly decreased after occlusion; however, they gradually recovered at later time points. Nevertheless, the reduction of a- and b- waves continued for more than 30 days, compared to only 3 days of retinal dysfunction determined by ERG in a previously reported experimental RVO model, due to recanalization^19^. This recanalization is a serious limitation of the previous model. In our model, the decrease in blood flow continued until day 30 and impairment of visual function remained apparent after 30 days. Interestingly, the b/a amplitude ratio of the ERG was significantly decreased in the RVO model on days 1 and 3. Reduction of the b/a wave amplitude ratio is an indicator of inner layer dysfunction and a characteristic feature of ischemic retinal disorders, such as diabetic retinopathy and RVO^32^. These results suggest that the inner retina is more impaired than the outer retina by retinal vein occlusion in our RVO model, resulting in dysregulation of retinal homeostasis and retinal volume^30^,^33^. The b/a wave amplitude ratio gradually improved over the course of the experiment, mainly due to the recovery of the amplitude of the b-wave. In addition, the thickening of the INL was also ameliorated in time-dependent manner. Furthermore, the expression level of *Aqp4* increased remarkably 7 days after occlusion. The amplitude of the b-wave is reduced in aquaporin knockout mice compared to wild type controls^34^. Immunostaining revealed that the expression of Aqp4 was significantly decreased in the INL (Supplemental Fig. 4). Together, these data indicate that alteration of *Aqp4* expression is strongly associated with retinal function; therefore, it is possible that the improvement in INL thickness depends on the recovery of the function of Müller cells, meditated by alterations in *Aqp4* mRNA expression. In contrast, the amplitude of the a-wave was most notably decreased on day 7. In the present experimental model, retinal detachment was commonly observed, occurring in approximately 90% of treated eyes. Previous clinical reports suggest that RVO patients with serous detachment have a reduced or absent ellipsoid zone, which is an indication of photoreceptor cell death, resulting in impairment of visual function^35^. Moreover, the thickness of the ONL and OPL was reduced in rat RVO and experimental retinal detachment models^36^,^37^. Hence, the reduction of the a-wave observed on day 7 was probably a consequence of retinal detachment.

Retinal nonperfusion is an important finding in RVO, as there is a strong correlation between the area of retinal nonperfusion and the severity of macular edema^3^,^38^. Therefore, we evaluated the area of retinal nonperfusion using retinal flat-mounts in the present experimental RVO model. We found that retinal nonperfusion occurred 1 day after occlusion and that the area of nonperfusion reduced over time. Previous reports indicate that retinal neovascularization occurs after the formation of capillary retinal nonperfusion^39^ and the decrease in the size of the nonperfused area of the retina indicates that neovascularization has occurred. The severity of both of retinal edema and retinal nonperfusion were the most significant on day1. There was a positive correlation between the severity of macular edema detected in OCT images and size of the nonperfused area of the retina^3^. Furthermore, the size of the nonperfused retinal area and the severity of macular edema depend on the expression of VEGF and IL-6^38^. In our model, expression of both *Vegfa* and *Il6* were notably increased 1 day after occlusion compared to untreated eyes, and expression of both genes gradually reduced at same time point (day 3), consistent with the observed improvement in edema and retinal nonperfusion, albeit *Il6* expression was up-regulated on day 7. Further, we investigated the expression of inflammatory genes (*Il-6, Mcp-1, Icam-1*) and *Vegfa* mRNA in sham-operated mice (Supplemental Fig. 3). These genes were not altered compared to normal 0.5, 1, 3, and 7 days after laser irradiation. Moreover, edema was not formed in sham-operated mice. These data indicated that the formation of edema was caused due to the up-regulation of *Vegfa* and *Il-6* elicited by occlusion of retinal vessels although we cannot completely exclude the effects of laser induced inflammation. Next, we performed long-term observation of the ocular fundus and retinal layers. No previous reports of RVO models detail observations over 30 days, indicating that only acute pathology was investigated in those studies, despite the fact that RVO is a chronic disorder^13^,^14^. In our model, white flecks and hyper-reflective dots were observed in the INL and OPL on day 30. These deposits consisted of hard exudates, including lipoproteins and other proteins, leaking from retinal blood vessels and are formed after resorption of intra-retinal edema. Hard exudates are commonly examined by PAS stain in clinical practice. In the present experimental model, some deposits in the INL and OPL were stained by PAS stain, confirming that they were hard exudates. Although we performed some experiments over 30 days, we cannot judge whether “day 30” is equivalent to the chronic phase of human RVO, because the speed of pathological progression in animal models is different to that in patients. However, we can use this model to investigate the effects of some pharmaceutical candidate compounds on retinal dysfunction and nonperfused retina because these symptoms were kept over 14 days. Hence, although our model also has some of the same limitations as previous models, we have established an improved and more suitable RVO model, with a number of characteristics more reminiscent of clinical features than those of previous models. There are two main differences between the present model and previous models: (1) the relevant mouse strain and (2) the dye used. Previous studies have used C57BL/6J and Balb/c mouse strains^26^,^27^. We attempted to generate RVO models using these strains; however, we were unable to establish a model with the feature of edema. One previous RVO model was established after fluorescein injection; however, we confirmed that spontaneous recanalization was observed 3 days after irradiation when using fluorescein. In another model, rose bengal was used; however the concentration used was lower than that employed in the present study and we confirmed that spontaneous recanalization was observed after use of rose bengal at the lower concentration used in the previous study (Supplemental Fig. 2). Hence, both the specific mouse strain employed and the type and concentration of dye used are important differences between this model and previously reported models.

Next, we investigated the effect of anti-VEGF antibody on retinal edema and retinal nonperfusion. There are no previous reports of the use of mouse anti-VEGF antibody to validate RVO animal models and investigate whether edema is induced via the VEGF pathway. There are some reports using bevacizumab^14^,^40^,^41^; however, bevacizumab influenced the immune systems of the study animals, since it is a humanized antibody^14^. This limitation has meant that these models have not been used to explore novel treatments. As expected, the development of edema was ameliorated by administration of mouse anti-VEGF antibody, indicating that our experimental model has similar features to those observed in clinical practice, including the development of edema via the VEGF pathway.

Moreover, the anti-VEGF antibody ameliorated retinal nonperfusion in the early phase after occlusion, although administration at a later time point resulted in deterioration, consistent with conflicting previous reports of opposite effects^6^,^7^,^8^,^9^. The timing of administration of the anti-VEGF antibody may be important in respect of its effects on retinal nonperfusion. To test this hypothesis, anti-VEGF antibody was administrated immediately and 7 days after occlusion. Administration immediately after occlusion reduced the size of the nonperfused retinal area, possibly as a result of a reduction in tissue pressure. Neutralization of ischemia-induced VEGF signalling reduces edema and decreases tissue pressure, resulting in an improvement of ocular circulation and a reduction in the nonperfused retinal area. This improvement of nonperfused retina seems to be caused by reduction of leukocyte adhesion which can induce blood flow abnormality. Leukocyte adhesion was commonly seen in patients with retinal vascular disorder such as diabetic retinopathy and patients with human immunodeficiency virus infection, leading to blood flow abnormalities^42^,^43^. This abnormality may promote the enlargement of retinal nonperfused area. The previous report suggested that VEGF promoted leukocyte adhesion to endothelial cells^44^. Although there is no report to indicate the relationship between blood flow abnormality and leukocyte adhesion in RVO patients, the previous report suggested that leukocyte rolling was more frequency in retinal veins rather than in arteries^45^. Moreover, we have elucidated that ICAM-1 which is strongly associated with leukocyte adhesion was increased in this experimental model but further consideration will be needed to clarify the role of leukocyte in present RVO model and the effects of anti-VEGF antibody on leukocyte adhesion. In addition, anti-VEGF antibody has another effect such as a vasoconstrictor. VEGF promote the synthesis of nitric oxide (NO) via increase of the endothelial cell NO synthase, causing vasodilatation^46^. Anti-VEGF antibody involves the reduction of produced NO and vasoconstriction. In our experimental model, the size of nonperfused retinal area was enlarged in later phase by anti-VEGF antibody. This aggravation may be caused by inhibition of synthesis of NO.

In conclusion, the RVO model established in this study more closely mimics the clinical manifestations of RVO, with regard to cystoid edema, retinal haemorrhage, retinal nonperfusion, and hard exudates, without inducing collateral damage. This model will be a valuable and useful tool to investigate the effects of anti-VEGF antibody and develop preclinical drug targets without the need for extremely large clinical trials. Moreover, our study demonstrates that the timing of anti-VEGF antibody administration is strongly associated with differences in progression and improvement of retinal nonperfusion, although further studies are required to elucidate the mechanisms underlying the opposing effects exerted by anti-VEGF antibody.

## Material and Methods

### Study Approval

All investigations were performed in accordance with the ARVO statement for the Use of Animals in Ophthalmic and Vision Research, and the experiments were approved and monitored by the Institutional Animal Care and Use Committee of Gifu Pharmaceutical University.

### Animals

We purchased ddY (8 weeks old), C57Bl/6J (7 weeks old), and BALB/c (7 weeks old) male mice from Japan SLC (Shizuoka, Japan), Jackson Laboratory (Bar Harbor, ME, USA), and Charles river (Barcelona, Spain), respectively. The animals were housed at 23 °C ± 3 °C, under 12 h light/dark cycles (lights on from 08:00 to 20:00).

### RVO model

Mice were anesthetized by intramuscular injection of a mixture of ketamine (120 mg/kg; Daiich-Sankyo, Tokyo, Japan) and xylazine (6 mg/kg; Bayer, Health care Osaka, Japan). Rose bengal (8 mg/mL) (Wako, Osaka, Japan) was injected into a tail vein, and 10 to 15 laser spots per vein (three veins per animal) were applied to a branch vein (3 disc diameters from the optic nerve centres) on the right eye of each animal using an image-guided laser system (532 nm) attached to a Micron IV Retinal Imaging Microscope (Phoenix Research Laboratories, Inc., Pleasanton, CA, USA) at 50 mW power (duration: 5,000 ms; spot size: 50 μm).

### Image Guided 830 nm Optical Coherence Tomography imaging

OCT images were taken 1, 3, 7, 14, 30, 90, and 180 days after laser photocoagulation, as were corresponding OCT scans using a Micron IV fundus camera and an OCT Scan Head equipped with a mouse objective lens (Phoenix Research Labs, Pleasanton, CA, USA). The OCT device featured a broadband superluminescent diode at 830 nm, customized for retinal imaging of mice. The scan region on the mouse retina was 1.8 mm in the X and Y directions. Linear OCT scans consisted of a series of 1024 single point A-Scans. Right eyes had previously been dilated with 0.5% tropicamide (Santen Pharmaceutical Co. Ltd., Osaka, Japan) and hydroxyl ethyl cellulose (Senju Pharmaceutical Co. Ltd., Osaka, Japan), used as coupling gel. Images were captured from 20 positions for each eye using StreamPix 6 and Micron OCT commercial software (Phoenix Research Labs). Captured images were quantitatively analysed using “In Sight” software, which can automatically detect and measure each retinal layer. Using this software, the retinal thickness was measured at 20 recorded positions for each eye, and the average of all positions represented the overall retinal thickness.

### Ocular Fundus Photography

Ocular fundus images were obtained using a Micron IV Retinal Imaging Microscope. Ocular fundus photography using this microscope is a simple, non-invasive technique for retinal imaging that can be used to evaluate adaptive changes in retinal vasculature. Five microliters of ophthalmic solution, containing 0.5% tropicamide and 0.5% phenylephrine hydrochloride (Mydrin-P; Santen) was applied topically after anaesthesia to dilate the pupil, and then mice were anesthetized using ketamine and xyladine. A few minutes later, hydroxyethyl cellulose gel (Scopisol; Senju) was applied topically to prevent desiccation and to keep the surface smooth. All fundus images were captured at the same time as OCT images.

### Electroretinography

Electroretinography (ERG) measurement was performed as described previously^47^, at 1, 3, 7, 14, and 30 days after laser photocoagulation. All procedures were performed in dim red light, and mice were kept warm during the entire procedure. The amplitude of the a-wave was measured from the baseline to the maximum a-wave peak, and the b-wave was measured from the maximum a-wave peak to the maximum b-wave peak.

### Histological Analysis

To visualize histological changes, we performed H&E and PAS staining of mouse eye sections. Mice were euthanized by cervical dislocation under deep anaesthesia, and each eye was enucleated. Eyes used for histological analysis were kept immersed for at least 48 h at 4 °C in a fixative solution containing 4% paraformaldehyde. Six paraffin-embedded sections (thickness, 5 μm) were cut parallel with the maximal circumference through the optic disc. Eye sections were then prepared in the standard manner, and stained with H&E and PAS. The damage induced by retinal vein occlusion was then evaluated, using three H&E-stained sections from each eye for morphometric analysis. Light-microscope images were photographed, and the INL and ONL from the optic disc were measured in the photographs at 240 μm intervals.

### Blood flow measurement with Laser Speckle Flowgraphy

A laser speckle flowgraphy (LSFG; Softcare, Iizuka, Japan) device was used as previously described with slight modifications to measure the blood flow. This device consists of a fundus camera equipped with a halogen lamp and a diode laser (λ = 830 nm, maximum output power, 1.2 mW). The measured fundus area was approximately 3.8 × 3 mm (width × height), with an estimated depth of tissue penetration of 0.5 to 1 mm. A speckle pattern appears due to random interference of the scattered light from the illuminated area, which was imaged by an instrument (700 × 480 pixel) at a frequency of 30 frames/s. Offline analysis software was used to compute the mean blurring rate (MBR), which is closely associated with blood flow velocity. The MBR was then extracted with a software tool.

### RNA extraction and real-time PCR

To examine time-dependent changes in RVO related gene expression after laser photocoagulation, non-treated and laser irradiated retinas were obtained 0.5, 1, 3 and 7 days after photocoagulation. Mice were euthanized by cervical-spine dislocation, and the eyeballs quickly removed. Retinas were carefully separated from the eyeballs and rapidly frozen in liquid nitrogen. RNA was isolated from retinas with the aid of a High Pure RNA Isolation kit (Roche Diagnostics, Tokyo, Japan). RNA concentrations were determined spectrophotometrically at 260 nm. First-strand cDNA was synthesized in 10 μL reaction volumes using a PrimeScript RT reagent kit (Perfect Real Time; Takara Bio Inc., Shiga, Japan). Gene expression was quantified by means of real-time PCR, performed using SYBR Premix Ex Taq (Takara) and a TP 8000 Thermal Cycler Dice Real Time system (Takara). The PCR primer sequences of *Vegfa, Il6, Mcp-1, Icam1, Pdgfa* and *Aqp4* were as follows: *Vegfa*, 5′-ACATTGGCTCACTTCCAGAAACAC-3′ (forward) and 5′-GGTTGGAACCGGCATCTTTATC-3′ (reverse); *Il6*, 5′- TCTGCAAGAGACTTCCATCCAGT -3′ (forward) and 5′-TCTGCAACTGCATCATCGTTGT-3′ (reverse); *Mcp-1*, 5′-CTGAAGCCAGCTCTCTCTTCCT-3′ (forward) and 5′-CAGGCCCAGAAGCATGACA-3′ (reverse); *Icam1*, 5′-CGCTGTGCTTTGAGAACTGTG-3′ (forward) and 5′-ATACACGGTGATGGTAGCGGA-3′ (reverse); *Aqp4*, 5′-GAGTATGTCTTCTGTCCTG-3′ (forward) and 5′ACGGTCAATGTCAATCAC-3′ (reverse); *Pdgfa*, 5′-GTCCAGGTGAGGTTAGAGG-3′ (forward) and 5′-CACGGAGGAGAACAAAGAC-3′ (reverse); and *Glyceraldehyde-3-phosphate dehydrogenase (Gapdh*) 5′-GGGATGGTCCTTGCATCAGAA-3′ (forward) and 5′-ACTGGTAGCCACTGGTCTGGTTG-3′ (reverse).

### Imaging of retinal nonperfusion

Mice were sacrificed at 1, 3, 7, and 30 days after photocoagulation and 0.5 mL of 20 mg/mL fluorescein isothiocyanate-dextran (Sigma–Aldrich, St. Louis, MO, USA) dissolved in PBS was injected into tail veins. Eyes were enucleated and fixed for 7 h in 4% paraformaldehyde, and retinal flat-mounts were prepared. Total images of flat-mounted retinas were produced using Metamorph (Universal Imaging Corp., Downingtown, PA, USA). To evaluate the retinal avascular area, we used image processing software (ImageJ ver. 1.43 h; National Institutes of Health, Bethesda, MD, USA).

### Drug administration

Mouse anti-VEGF antibody (200 μg/mL; R&D Systems, Minneapolis, MN, USA) was used to confirm whether VEGF is involved in the development of edema or retinal nonperfusion in our experimental RVO model. Immediately or 7 days after laser occlusion, mice received an intravitreal injection of anti-VEGF antibody in their right eyes. Sterile 34-gauge needles (Terumo, Tokyo, Japan) were attached to fine-bore tubes (Natsume Seisakusho, Tokyo, Japan) and filled with the anti-VEGF antibody. A microsyringe was connected to the opposite side of the tube. Under microscopic guidance, the limbus of the cornea was pierced toward the posterior segment with a needle, and then 2 μL of the anti-VEGF antibody was administered into the vitreous cavity. After intravitreal injections, the sclera was disinfected with 0.5% levofloxacin (Santen).

### Statistics

Statistical analyses were performed using the Statistical Package for the Social Sciences 15.0 J for Windows software (SPSS Japan Inc., Tokyo, Japan). Data are presented as means ± SE. Statistical comparisons were conducted using Student’s *t*-test, Dunnett’s multiple comparison test, or one-way ANOVA followed by Bonferroni’s post hoc comparison test. P values < 0.05 were considered statistically significant.

## Additional Information

**How to cite this article**: Fuma, S. *et al*. A pharmacological approach in newly established retinal vein occlusion model. *Sci. Rep.* **7**, 43509; doi: 10.1038/srep43509 (2017).

**Publisher's note:** Springer Nature remains neutral with regard to jurisdictional claims in published maps and institutional affiliations.

## Supplementary Material

Supplementary Figure
