# Supplementary material for: A pharmacological approach in newly established retinal vein occlusion model
Source: Sci Rep. 2017 Mar 2;7:43509. doi: 10.1038/srep43509 (PMC5333144; doi:10.1038/srep43509)
Supplement: Supplementary Figure [file srep43509-s1.pdf]

For submission to Scientific Reports

A pharmacological approach in newly established retinal vein occlusion model

Shinichiro Fuma[1], Anri Nishinaka[1], Yuki Inoue[1], Kazuhiro Tsuruma[1], Masamitsu Shimazawa[1], Mineo Kondo[2], Hideaki Hara[1] *

[1]Molecular Pharmacology, Department of Biofunctional Evaluation, Gifu Pharmaceutical University, Gifu, Japan.

[2]Department of Ophthalmology, Mie University Graduate School of Medicine, Tsu, Japan.

***Correspondence to:** Prof. Hideaki Hara, Ph.D., Molecular Pharmacology, Department of Biofunctional Evaluation, Gifu Pha47rmaceutical University, 1-25-4 Daigaku-nishi, Gifu 501-1196, Japan; Phone/Fax: +81-58-230-8126; e-mail: hidehara@gifu-pu.ac.jp

Supplemental figure

Materials and Methods

Immunostaining

The enucleated eyes were fixed in 4% paraformaldehyde for 48 h at 4°C, soaked in 25% sucrose for 24 hours at 4°C, and embedded in optimum cutting temperature (OCT) compound (Sakura Finetechnical Co., Ltd., Tokyo, Japan). These tissues were immediately frozen with liquid nitrogen and stored at −80°C. Serial transverse sections were cut on a cryostat to a thickness of 10 μm and placed on slides (MAS COAT; Matsunami Glass Ind. Ltd., Osaka, Japan) for immunohistochemistry. Immunohistochemical staining was performed in accordance with the following protocol. Briefly, tissue sections were washed in 0.01 M PBS for 5 minutes, followed by preincubation with 10% normal goat serum. Anti-aquaporin-4 antibody (1:2000, Sigma) was applied to the sections overnight at 4°C. Then, these sections were incubated for 1 h with Alexa 488 (Invitrogen Japan K.K., Tokyo, Japan). At the end of immunostaining, Hoechst 33342 (1:1000) was added to the samples for 5 minutes. The fluorescent images were taken with Metamorph (Universal Imaging Corp., Downingtown, PA, USA).

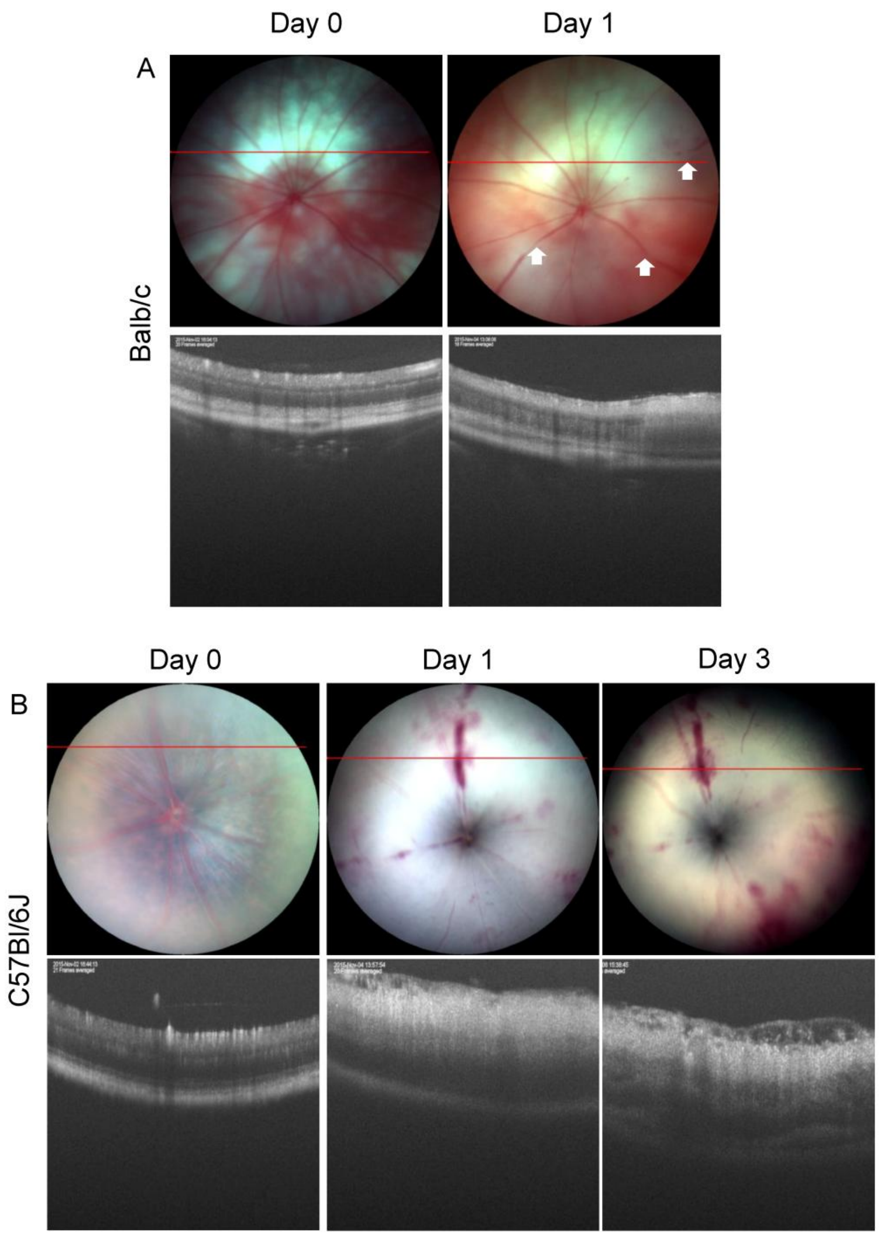

Supplemental figure 1: Ddy mice were more suitable to create RVO model than balb/c and C57BL/6J mice.

In order to clarify the causes why the edema was observed in our model using ddy mice whereas other established RVO models did not have edema, balb/c and C57BL/6J mice which were used in previous RVO mice models were occluded by laser irradiation. Edema was not formed in C57BL/6J mice and the incidence of retinal hemorrhage was lower than ddy mice

although these mice were irradiated under same conditions as ddy mice. Furthermore, early recanalization after only 1 day was observed. In C57BL/6J mice, edema was formed 3 days after occlusion, whereas it was formed 1 day after occlusion in ddY mice. Unfortunately, some arteries were occluded by laser irradiation despite the fact that we irradiated only retinal under the same conditions as ddY mice.

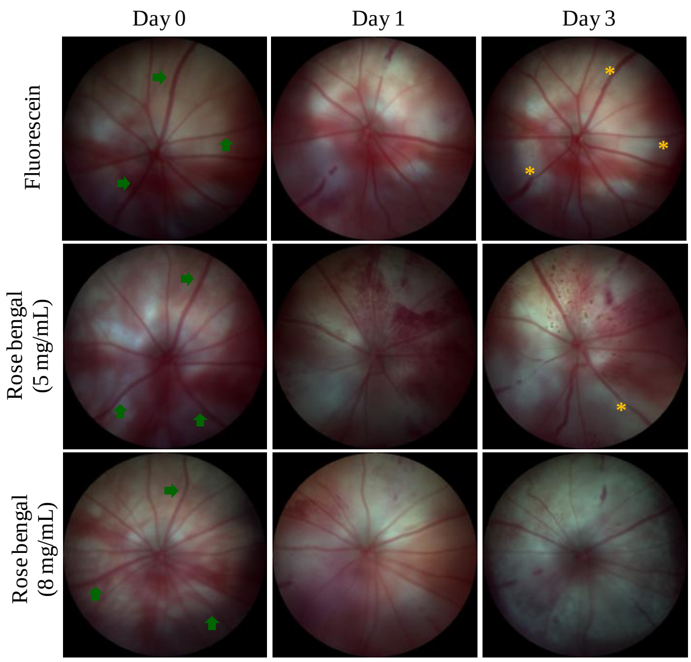

Supplemental figure 2: Spontaneous recanalization was observed in mice with fluorescein or lower concentration of rose bengal within only 3 days.

Mice were divided into 3 groups, 8mg/mL rose bengal injected mice, 5 mg/mL rose bengal injected mice, and fluorescein injected mice. Spontaneous recanalization was observed within 3 days in both fluorescein-injected mice and rose bengal (5 mg/mL)-injected mice while occlusion was kept 3 days after irradiation in mice with rose bengal (8 mg/mL). Green arrows indicate the site of laser irradiation and yellow arrows show the site of recanalization.

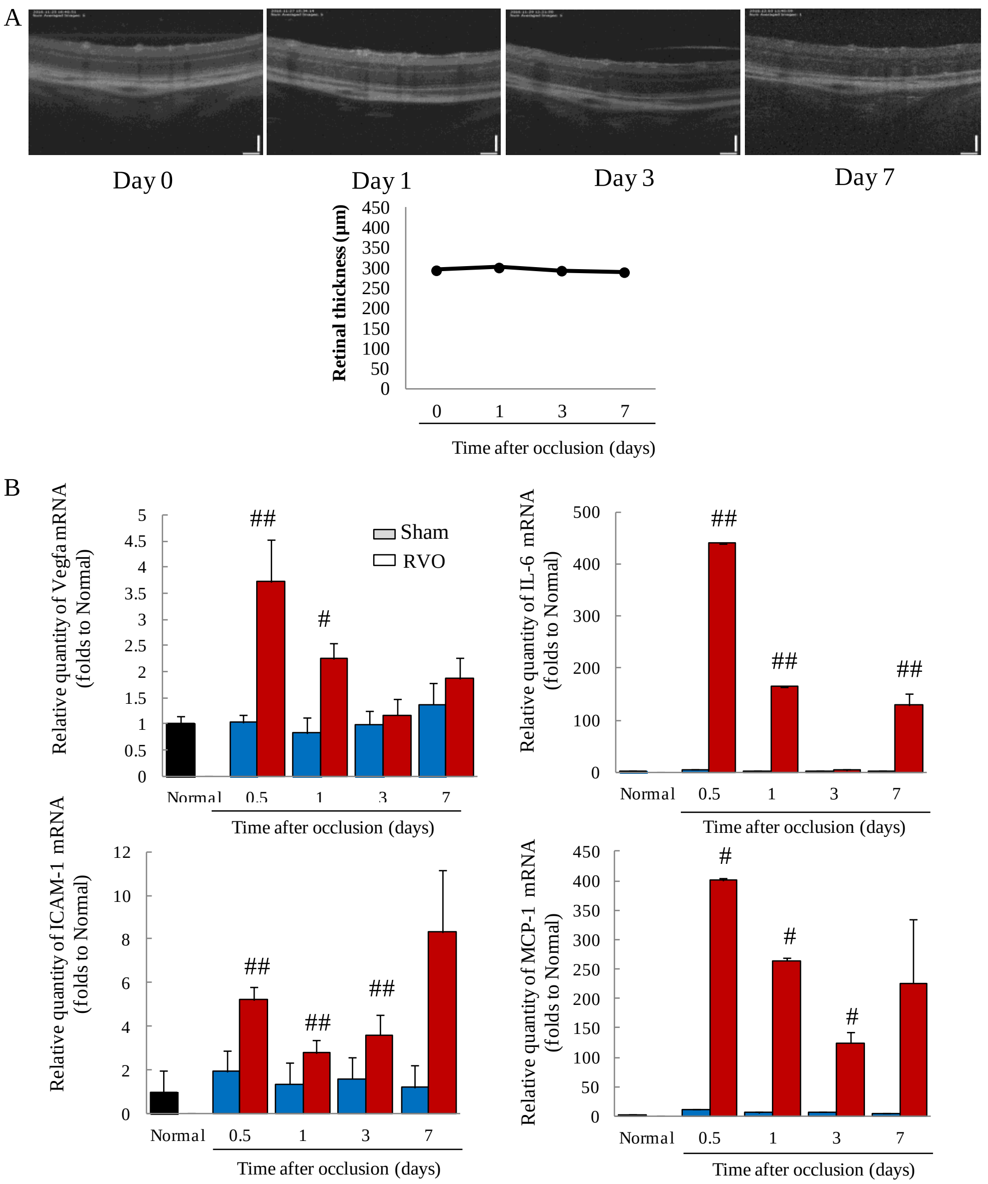

Supplemental figure 3: Edema was not formed by laser-induced inflammation.

To verify the effects of laser irradiation regarding formation of edema, we used sham-operated mice. (A) Images are representative OCT images of sham-operated mice and the below is the quantitative data of the thickness of retina. The thickness of retina did not change in sham-operated mice. (B) The expression of VEGFA and inflammatory genes were evaluated by real-time PCR analysis in sham and RVO mice. The expression of VEGFA and inflammatory mRNA in sham-operated mice did not change.

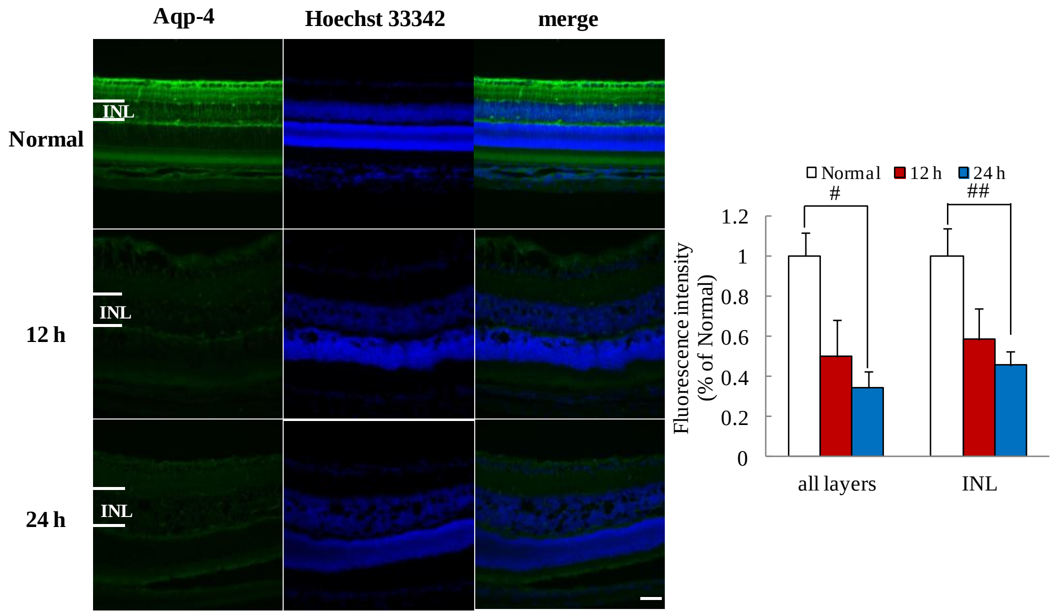

Supplemental figure 4: The expression of aquaporin-4 was decreased in retina, especially in INL

In order to clarify the expression and localization of aquaporin-4, we performed immunostaining. Representative images and quantification data of aqp-4. The expression of aqp-4 was decreased 12 and 24h after occlusion in retina and INL. Scale bar = 50 µm. Data are expressed as means ± S.E.M (n = 3-6). ##P < 0.01 , #P < 0.05vs. day 0 (Dunnett's test).
